# Own-Race Faces Capture Attention Faster than Other-Race Faces: Evidence from Response Time and the N2pc

**DOI:** 10.1371/journal.pone.0127709

**Published:** 2015-06-04

**Authors:** Guomei Zhou, Zhijie Cheng, Zhenzhu Yue, Colin Tredoux, Jibo He, Ling Wang

**Affiliations:** 1 Department of Psychology, Sun Yat-sen University, Guangzhou, Guangdong, China; 2 Department of Psychology, University of Cape Town, Cape Town, Western Cape, South Africa; 3 Department of Psychology, Wichita State University, Wichita, Kansas, United States of America; 4 Center for Studies of Psychological Application & School of Psychology, South China Normal University, Guangzhou, Guangdong, China; Bournemouth University, UNITED KINGDOM

## Abstract

Studies have shown that people are better at recognizing human faces from their own-race than from other-races, an effect often termed the Own-Race Advantage. The current study investigates whether there is an Own-Race Advantage in attention and its neural correlates. Participants were asked to search for a human face among animal faces. Experiment 1 showed a classic Own-Race Advantage in response time both for Chinese and Black South African participants. Using event-related potentials (ERPs), Experiment 2 showed a similar Own-Race Advantage in response time for both upright faces and inverted faces. Moreover, the latency of N2pc for own-race faces was earlier than that for other-race faces. These results suggested that own-race faces capture attention more efficiently than other-race faces.

## Introduction

Extensive research has provided evidence that people process own-race faces better than other-race faces (see [1] for a meta-analytic review). The Own-Race Advantage (ORA) effect (also known as the own-race effect, own-race bias, or in-group advantage, see [2]) has been traditionally found in face recognition tasks [1], and recently was found in a configural processing task [3] as well as in a feature processing task [4]. Some researchers are concerned about whether there is an ORA in attention, but the evidence is not consistent and the underlying mechanisms are not clear.

Some research shows that own-race faces capture attention more efficiently than other-race faces. For example, Hodsoll et al. [5] investigated whether the preferential allocation of attention to infant faces was influenced by the race of the faces and of the perceivers, by using a dot-probe task. Their data showed that participants responded faster to infant faces that appeared in the same location as own-race faces than other-race faces. Golby et al. [6] asked participants undergoing functional MRI to view own-race faces, other-race faces and objects, and to press a button to indicate their attention to the stimuli. They observed that both the right and left fusiform gyrus activated more strongly in response to own- versus other-race faces (see Natu et al. [7] for a review of neuroimaging studies of ORA effect).

Some researchers found an ORA with a change blindness task [8] [9]. Humphreys et al. [8] asked White Caucasian and Indian Asian participants to view scenes involving White Caucasian and Indian Asian students. Some parts of the scenes changed, such as the faces of two races, the bodies of two races, or an independent object in the background. Results showed that participants detected changes in own-race faces faster than they detected changes in other-race faces, but there was no difference in the detection of body changes. The authors suggested that it was not an effect of attention but an effect of sensitivity. The authors argued that attention was paid to the whole person. If the faces of own race attracted more attention, the bodies of that race should also attract more attention. Therefore, the results indicated that people were more sensitive to own-race faces. To replicate the study of Humphreys et al. [8], Hirose et al. [9] measured eye movements of Caucasian and Asian participants with the same materials. Similar to Humphreys et al. [8], their results showed quicker detection for own-race faces, but equally attention to own race faces and other race faces in terms of fixation order, number and duration. The results support the proposition that people are more sensitive to changes made to own-race faces [9].

Other studies even show an opposite effect of the own-race advantage for attentional processes, i.e., people may have an attentional preference for other-race faces [10] [11]. For example, Levin [10] asked White participants to search for White faces among Black faces or search for Black faces among White faces, and found that Black targets were detected faster than White targets by White participants. In several other gender/racial categorization tasks using the ERP technique, attention was first directed to other-race faces while later relocated to own-race faces, i.e. Black targets evoked larger early selective attention related components (N1 and P2 waves) than did White targets, while White targets evoked larger N2 waves compared with Black targets [12–14].

The mixed results of the above-mentioned studies could possibly result from different processing levels required by the tasks. The *categorization-individuation model* (CIM) [15] [16] suggests that perceivers have a ubiquitous tendency to think categorically about out-group members while processing the detailed individual facial features of in-group members (i.e., individuation or individual processing). It is likely that people detect other-race faces more quickly in the race-searching task [10] and the race categorization task [12–14], because these tasks require participants to categorize race of faces. People may detect own-race faces quicker in the change detection task [8] [9], which requires processing at the individual level. Other studies have also suggested that there is an other-race advantage in the race category processing (e.g., to judge the race of a target face) [17] [18] and an Own-Race Advantage in the individuation processing (e.g., to identify a target face) [1] [18–20]. Therefore, the other-race advantage in the race-searching task [10] may be a result of a categorization effect (the tendency to categorize other-race faces) or an attention effect (other-race faces capture attention more easily). The own-race advantage in change detection tasks [8] [9] might result from the individuation effect (a tendency to individuate own-race faces, or greater sensitivity to changes to own-race faces) or an attention effect (paying more attention to own-race faces results in being more easily able to detect changes to own-race faces). That is, the effect of attention may be masked or counteracted by other effects, such as the processing level. The reason that previous studies reported mixed results is probably that they could not extract a unique effect for attention. Thus, whether there is an own-race advantage in attention, at least in part, remains unclear.

To examine whether own and other-race faces differentially capture attention, we should find a way to rule out potential confounding factors, e.g. processing level. We conjecture that a human face detection task (e.g., to search or identify a human face among animal faces) does not need racial categorization or individuation processing; asking participants to detect human faces among animal faces is an effective way to get at the unique effect of attention.

Experiment 1 employed a visual search paradigm as previously used by Levin [10] except that we instructed participants to judge whether human faces were present in a particular set of stimuli, instead of performing a race-searching task. If there is an own-race advantage in attentional capture, we expected to observe faster detection of own-race faces than other-race faces when these were placed among animal faces. Experiment 2 was designed to explore the neural correlates of ORA in a human face detection task.

## Experiment 1

### Method

#### Participants

Twenty-one Chinese participants (9 males, 12 females between the ages of 19 and 32 years, *M* = 23 years, *SD* = 3 years) from Sun Yat-Sen University, and twenty-two Black African participants (11 males, 11 females between the ages of 18 and 28 years, *M* = 22 years, *SD* = 4 years) from the University of Cape Town were paid to participate in this experiment. All participants had normal or corrected-to-normal vision. The study was approved by the Human Research Ethics Committee of Department of Psychology in Sun Yat-Sen University and that in University of Cape Town. The participants all gave their written informed consent before taking part in the experiment.

#### Materials

**Fig 1:**
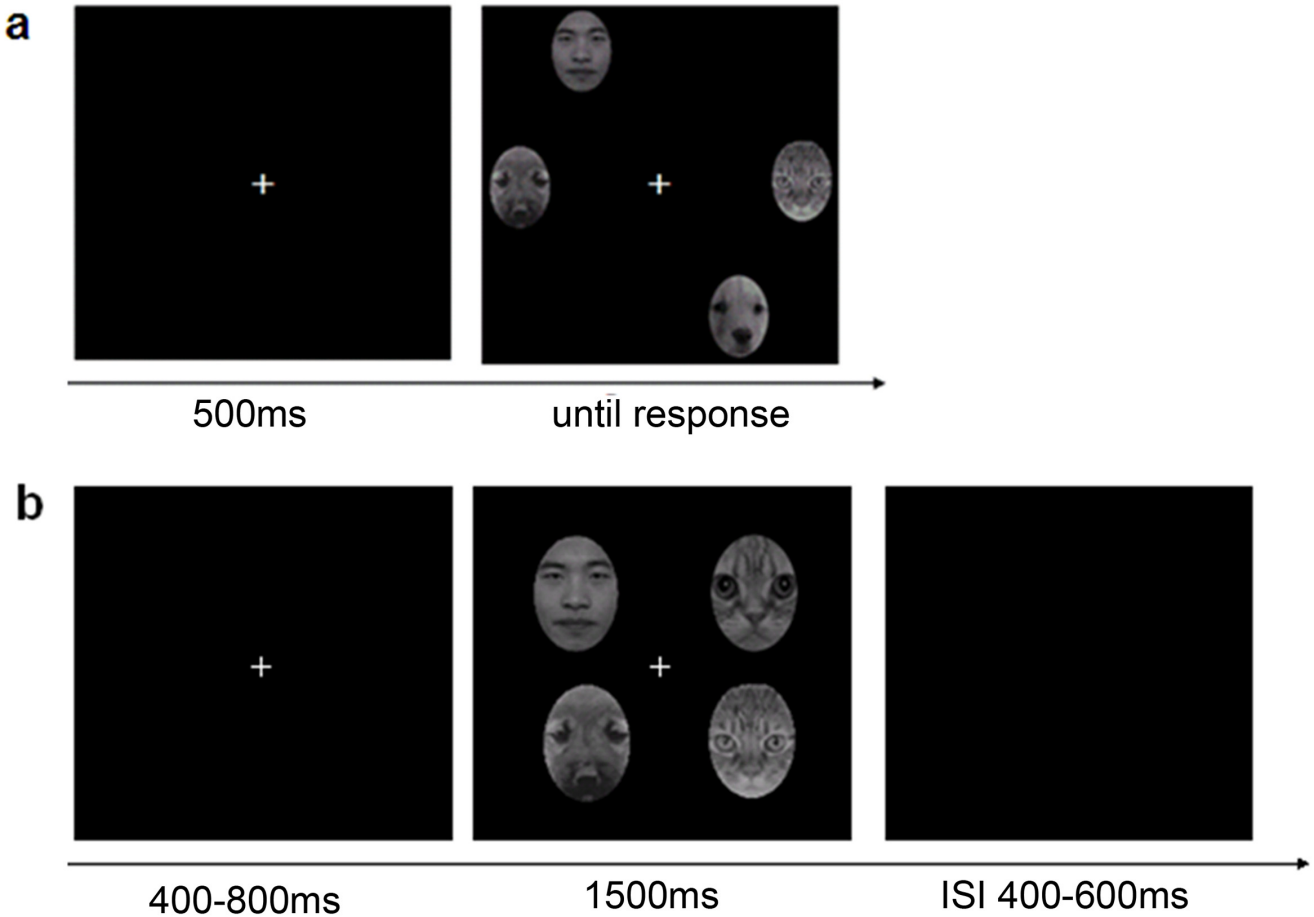
The trial procedures in Experiment 1 (a) and in Experiment 2 (b).

Photographs of three African American male faces [21] and three Chinese male faces collected in China were used as human faces. They were all grey-scale, front-view, digitally sectioned head shots with neutral facial expressions, with no glasses, beard, ears, or hair. Six animal faces served as distractors: three dogs and three cats. All these photos were digitally sectioned in the same way as the human faces. All faces were matched in luminance and contrast with Photoshop software; each subtended a visual angle of 1.7°×2.1° (see Fig 1a). The individual in Fig 1 has given written photo release form (as outlined in the PLOS consent form) to publish these case details.

#### Design

A 2 (Race of participants: Black or Chinese) × 2 (Race of human faces: Black or Chinese) × 3 (Set size: 2, 4, 6) mixed design was employed, with the Race of participants as a between-subjects variable.

#### Procedure

Participants were individually tested in a dimly lit room. The experiment was developed and executed with E-Prime (http://www.pstnet.com/eprime.cfm). All stimuli were presented at a viewing distance of 70 cm. Each trial began with a centrally presented fixation cross for 500 ms. Participants were asked to maintain gaze on the fixation cross, which was followed immediately by the search array, which remained on the screen until participants made responses. Then the next trial began. Half of the trials contained a target, and the other half of the trials contained no target face. In the target-present trial, one target human face (Black or Chinese) and 1, 3 or 5 animal faces (dogs' and cats' faces) were symmetrically positioned in one of the six positions around the central fixation point, with a radius of 5°. The number of animal faces was 1, 3 or 5, and the target face was equally likely to appear in the six possible positions. In the target-absent trials, no human faces were present, and there were 2, 4 or 6 animal faces on the screen. In each trial the participants were asked to search for the human face quickly and accurately, and respond using the keyboard by pressing the "f" key if a human face was present and the "j" key if no human face was present. We conjecture that such a human face detection task does not need racial categorization or individuation processing. Therefore, we used it to get at the unique effect of attention.

Participants completed nine practice trials before the formal test. The formal test included four blocks. In each block, half of the trials were target-present trials and half were target-absent trials; in the target-present trials, Black faces and Chinese faces served as targets with equal probability. All conditions were randomized. Each block contained 108 trials, and there were 432 formal trials in total.

### Results

#### Response time

**Fig 2:**
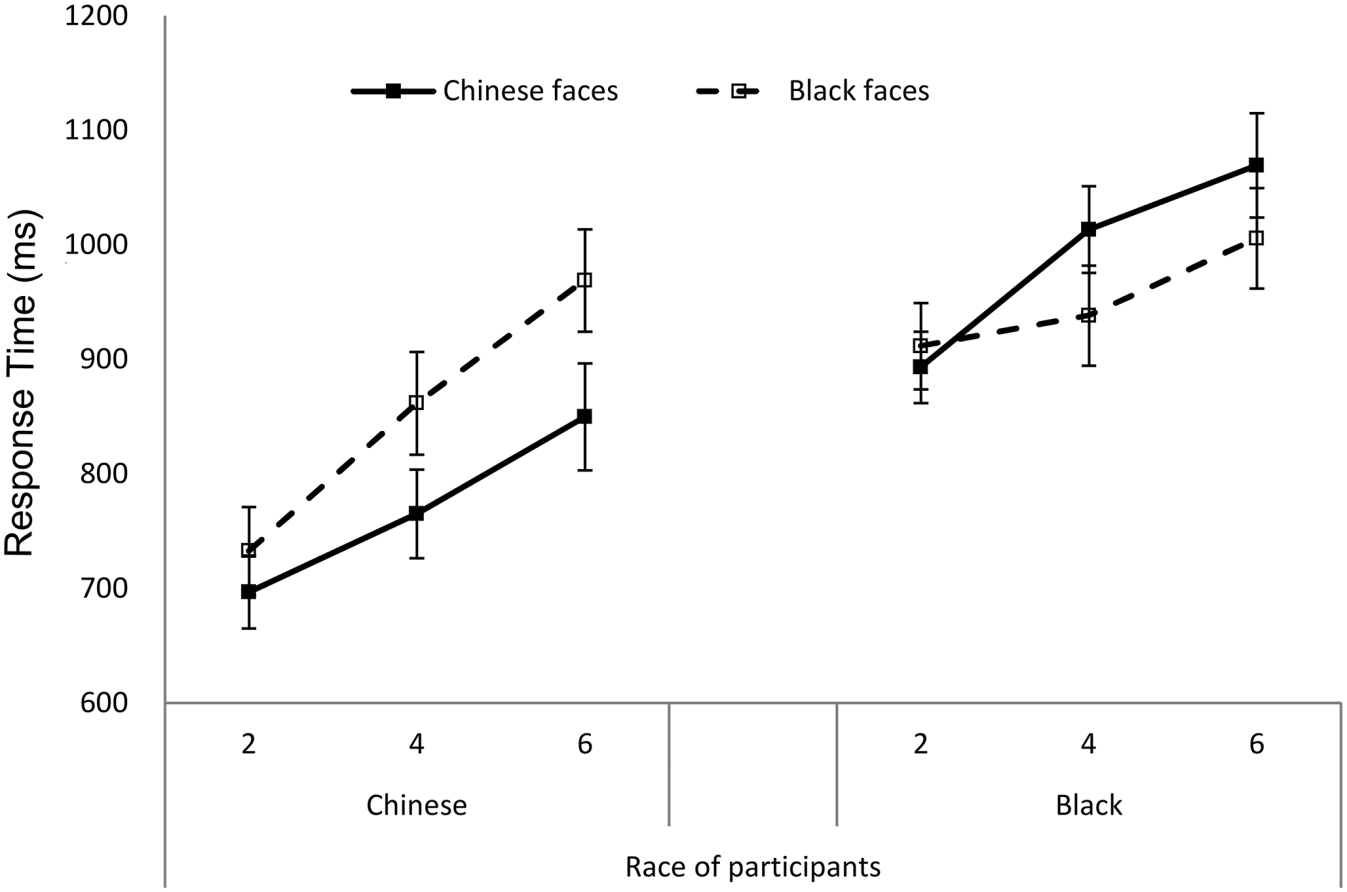
Response time as a function of Race of participants, Race of faces, and Set size in Experiment 1. The error bar represents the standard error.

Only correct human face responses were included in the analysis. Trials with response times (RTs) shorter than 100ms were discarded. Trials with RTs greater than 3 standard deviations from the mean of each condition were discarded (about 1.5% of all trials). Mean RTs for each participant were submitted to a mixed analysis of variance (ANOVA) with Race of faces (Black vs. Chinese) and Set size (2, 4, 6 items) as within-subjects variables and Race of participants (Black vs. Chinese) as a between-subject variable (see Fig 2).

There was a significant main effect of Race of faces, *F* (1, 41) = 4.89, *p* = .03, *η*_*p*_^*2*^ = .11; searching was faster when the target faces were Chinese faces (*M* = 812.54ms, *SE* = 38.58ms) than when they were Black faces (*M* = 971.88ms, *SE* = 37.69ms). Of interest was the significant interaction between Race of faces and Race of participants, *F* (1, 41) = 39.19, *p <* .001, *η*_*p*_^*2*^ = .49. Simple effect tests showed that for Chinese participants, searching human faces was faster for Chinese faces than for Black faces, *F* (1, 20) = 41.52, *p* < .001, *η*_*p*_^*2*^ = .68; for Black participants, searching human faces were faster for Black faces than for Chinese faces, *F* (1, 21) = 7.31, *p* = 0.013, *η*_*p*_^*2*^ = .26. There was a significant main effect of Set size, *F* (2, 82) = 89.71, *p* < .001, *η*_*p*_^*2*^ = .69, indicating that RTs increased with increasing set size. But the searching slope for Chinese subjects was steeper than that for Black subjects, with a marginally significant interaction of Set size and Race of participant, *F* (2, 82) = 2.93, *p* = .06, *η*_*p*_^*2*^ = .07. What is important is the significant three-way interaction, *F* (2, 82) = 6.06, *p* < .01, *η*_*p*_^*2*^ = .13. For Chinese participants, searching human faces was faster for Chinese faces than for Black faces, *F* (1, 20) = 41.52, *p* < .001, *η*_*p*_^*2*^ = .68. Race of faces significantly interacted with Set size, *F* (2, 40) = 8.42, *p* < .01, *η*_*p*_^*2*^ = .30. ORA was observed at all Set size conditions (*Fs*>14.73, *ps* < .001, *η*_*p*_^*2*^ *s*>.42). For Black participants, searching human faces was slower for Chinese faces than for Black faces, *F* (1, 21) = 7.31, *p* = .01, *η*_*p*_^*2*^ = .26. Race of faces interacted significantly with Set size, *F* (2, 42) = 5.91, *p* < .01, *η*_*p*_^*2*^ = .22. ORA was observed at Set size 4 and 6 conditions, *Fs*>5.68, *ps* < .05, *η*_*p*_^*2*^ *s*>.21, but not at the Set size 2 condition, *F* (1, 21) = 1.14, *p* = .30, *η*_*p*_^*2*^ = .05).

### Accuracy

The accuracy for all participants was above 80% (see Table 1). A mixed analysis of variance (ANOVA) showed a significant main effect of Race of faces, *F* (1, 41) = 15.74, *p* < .001, *η*_*p*_^*2*^ = .28, and its interaction with Race of participants, *F* (1, 41) = 34.48, *p* < .001, *η*_*p*_^*2*^ = .46. Overall, target human faces were detected more accurately for Chinese faces (*M* = 95.59%, *SE* = 0.75%) than for Black faces (*M* = 93.26%, *SE* = 0.72%) for all participants. A simple effect test indicated that this advantage was only observed for Chinese participants (*p* < .001), not for Black participants (*p* = .18). This result suggests that participants detected own-race faces faster without sacrificing accuracy. Other main effects and interactions did not reach significance (*p*s >.13).

**Table 1:** Means of accuracy (%) and standard errors (*SE*) in all conditions in Experiment 1.

		Set size					
		2		4		6	
		*Mean*	*SE*	*Mean*	*SE*	*Mean*	*SE*
**Chinese participants**	**Chinese faces**	95.29	1.37	97.19	1.19	95.86	1.18
	**Black faces**	91.38	1.05	89.48	1.79	90.10	1.67
**Black participants**	**Chinese faces**	94.41	1.34	94.91	1.17	95.91	1.16
	**Black faces**	96.64	1.02	95.91	1.75	96.05	1.63

### Discussion

We found a significant own-race advantage in a human face search task, which was inconsistent with the detection preference towards other-race faces in the race searching task reported by Levin [10]. The present ORA result was consistent with the own-race advantage in a task which requires individualization [18–20]. This provides a possibility that the own-race advantage in the current study comes from individual processing rather than attention. This possibility was further tested in Experiment 2.

A limitation of Experiment 1 is the small set of human face stimuli. As a consequence, it is very likely that idiosyncratic characteristics of the images or the depicted persons, and not race per se, drive the ORA effect. This should not be a very significant factor though, because the ORA was observed for both Chinese participants and Black participants, which indicates that the observed ORA effect was not related to the stimuli per se, but to the interaction of stimulus and participants’ race.

## Experiment 2

Experiment 2 was designed to explore the neural correlates of ORA in a human face detection task and to explore the underlying mechanism by recording N2pc (N2-posterior-contralateral), N170, and N250 waves.

First, we recorded N2pc to test whether the ORA in RT in Experiment 1 was related to attention. The *N2pc* wave, an attention-related ERP component, is used to measure the attentional processing of faces in the present study. The N2pc wave is typically elicited at posterior scalp sites contralateral to the position of a task-relevant visual stimulus between 200 and 300 ms after stimulus onset, as previously observed in experiments investigating the allocation of attention in visual search tasks [22–24]. The N2pc latency is correlated with rapid shifts of attention [25] [26]. If ORA in RT is related to the ability to capture attention (i.e. own-race faces capture attention more efficiently than other-race faces), searching for own-race faces would produce an earlier N2pc latency than searching for other-race faces.

Second, we recorded N170 to test whether the ORA in RT in Experiment 1 is related to categorization processing or configural processing. The N170 is a negative potential recorded around 170 ms after stimulus onset at posterior temporal sites, larger for faces compared with objects, and is believed to reflect face-specific configural processing [27–29] and has been used to measure race category experience (see [30] for a review). Studies using the categorization tasks found that upright own-race faces elicited larger [13] or lower [31] N170 amplitude than upright other-race faces. Although both studies used race categorization tasks, the former asked participants to indicate whether the face was Black or White, the latter required participants to identify whether a face belonged to their own race or not. However, studies using race-irrelevant tasks [32] [33] did not observe a race difference on N170 amplitude (see Wiese [34] for a review on how N170 varies with task demands). Therefore, if ORA in RT in Experiment 1 is related to race categorization, we would expect to observe a race difference in N170.

A configural processing advantage of the own-race faces could also contribute to the ORA effect [3] in the human search task. Inversion can impair the configural encoding of faces [35] [36], and is reflected in a latency delay and an amplitude increase of the N170 [27–29] (i.e., inversion effect). Therefore, Experiment 2 further examined the possible differences between races on configural processing of faces by adopting upright faces and inverted faces as stimuli. If the ORA in RT is related to configural processing, we would observe an inversion effect for own-race faces but not for other-race faces in RT and in N170.

Finally, we recorded N250 to testify whether the ORA in RT in Experiment 1 is related to individuation processing. N250 is a bilateral component that shows an ongoing negativity that peaks around 250 ms after stimulus onset, which measures the subordinate-level categorization or individuation (see [37] for a review). Several studies have found that other-race faces elicit more negative N250 than own-race faces [38] [39]. Compared with basic-level training, subordinate-level individuation training for other-race faces elicited an increased response in N250 component [40]. If ORA in RT is related to individuation processing, we would expect to observe a race difference in the N250.

### Method

#### Participants

Eighteen Chinese participants were paid to participate in this experiment. Two of them were excluded for eye movement artifacts or excessive alpha activity, leaving 16 participants (6 males, 10 females) between the ages of 19 and 27 years (*M* = 22 years, *SD* = 2.09), with normal or corrected-to-normal vision. One participant was left-handed, and others were right-handed. The study was approved by the Human Research Ethics Committee of Department of Psychology in Sun Yat-Sen University. The participants all gave their written informed consent before taking part in the experiment.

#### Materials

The experiment materials were exactly the same as those used in Experiment 1 except the following two differences: First, only four faces in a search array were used in the present experiment, given that the difference in detection time between Chinese faces and Black faces was large enough to show itself for a set size of four faces in Experiment 1. Second, inverted versions of human faces and animal faces were included in the present experiment to investigate whether there was configural processing involved.

#### Design

A 2 (Race of faces: Black, Chinese) × 2 (Orientation of faces: upright, inverted) within-subjects design was employed.

#### Procedure

Participants were seated 70 cm from the screen in a dimly lit room. The experiment was run in E-Prime (http://www.pstnet.com/eprime.cfm). Each trial (see Fig 1b) began with a centrally presented fixation point for 400–800 ms, followed immediately by a search array, which remained on screen for 1500 ms, containing four faces at a distance of 4° from central fixation. The inter-trial interval (ISI) jittered from 400–600 ms. In each trial participants were asked to search for the human face among animal faces as quickly and accurately as possible. If a human face was present, participants were required to press the number key “8”. If no human face was present, no response was needed. Participants were also instructed to keep fixating on the center of the screen during the trials.

Participants finished 16 practice trials before the formal test. The formal experiment was divided into 12 blocks, with half of the blocks for upright faces and the other half for inverted faces. The sequence of conditions was counterbalanced across participants. The target face was present in half of the trials; own-race faces and other-race faces were designated as the target with equal probability. Each block contained 96 trials. There were 1152 trials in total and 144 trials in each experimental condition. Participants were allowed to take a rest between blocks.

#### EEG recording and data preprocessing

Electroencephalogram (EEG) signals were recorded from 64-channel Ag/AgCl electrodes (ANT, Netherlands) mounted into an elastic cap according to the International 10–20 System. These electrodes were recorded using a right mastoid reference electrode, and the signals were re-referenced offline. Vertical eye movements were monitored (Electrooculogram, EOG) with electrodes above and below the left eye against the reference. Horizontal eye movements were recorded with two electrodes placed at the outer canthi of each eye. Impedance for each electrode was kept below 5 kΩ. For the N170, the average signal from all electrodes ('average reference') was used as reference. For the N2pc wave, all signals were digitally re-referenced offline to the average of the left and right mastoids. All recordings were amplified with a band pass of 0.01–100 Hz. Data were sampled at 512 Hz.

EEG analysis was performed with the assistance of the EEGLAB 6.0 toolbox. Raw data were band-pass filtered between 0.01 and 40 Hz. EEG was then segmented into epochs from 100 ms before the stimulus onset to 400 ms post-stimulus. Baseline correction was performed using the first 100 ms of the epoch. Trials with artifacts exceeding ±60μV in the horizontal and vertical EOG channels were eliminated. Trials that showed amplitudes exceeding ±100 μV for other channels were also eliminated. About 20–25% of trials were excluded on average due to artifacts. We also examined the averaged horizontal EOG waveforms after artifact rejection to ensure that small eye movements did not contaminate the average ERP waveforms. Incorrect responses and response time (RT) that were three standard deviations away from the mean in each experimental condition, which counted for about 1.7% of all trials, were excluded from final analysis. Thereafter, grand-averaged waveforms were calculated separately for all experimental condition combinations: Target race (Black, Chinese), Orientation (upright, inverted).

Mean amplitudes for N170 were measured at electrodes O1, O2, PO7, PO8, for the time window 180–230ms. Mean amplitudes for N250 were measured at these electrodes for the time window 260#x2013;320ms. The peak latency of N2pc was measured at the same four electrodes.

### Results

#### Response time

A repeated measures analysis of variance (ANOVA) with Race (Black, Chinese) and Orientation (upright, inverted) as within-subjects factors was calculated on mean response times. As in Experiment 1, the main effect of Race was significant, *F* (1, 15) = 8.07, *p* <.05, *η*_*p*_^*2*^ = .35. That is, Chinese participants searched human faces faster when the faces were Chinese (*M* = 638.67ms, *SE* = 30.66ms) than when they were Black (*M* = 652.80ms, *SE* = 28.50ms). Orientation produced a significant main effect, *F* (1, 15) = 37.36, *p* < .0001, *η*_*p*_^*2*^ = .71, with longer RT for inverted faces than for upright faces. The Race × Orientation interaction was not significant, *F* (1, 15) = 2.11, *p* = .17, *η*_*p*_^*2*^ = .12, indicating that upright and inverted faces showed the same own-race advantage pattern (shown in Table 2).

**Table 2:** Means of RTs (ms), means of accuracies (%), means of average amplitudes (μV) and peak latency (ms) of ERP and standard errors (*SE*) in all conditions in Experiment 2.

		Upright				Inverted			
		Chinese		Black		Chinese		Black	
		*Mean*	*SE*	*Mean*	*SE*	*Mean*	*SE*	*Mean*	*SE*
**RT**		596.42	30.10	619.21	32.03	680.93	32.76	686.38	26.25
**Accuracy**		99.25	0.56	98.75	0.79	98.31	0.68	98.50	0.75
**N170 amplitude**	O1	-2.25	1.71	-3.00	1.31	-1.50	1.53	-0.64	1.43
	O2	-1.13	1.57	-2.01	1.19	-0.26	1.44	-0.47	1.34
	PO7	-3.42	1.88	-4.28	1.47	-2.68	1.58	-1.74	1.50
	PO8	-1.77	1.78	-2.63	1.41	-0.69	1.51	-0.25	1.50
**N250 amplitude**	O1	-0.23	1.36	1.13	1.79	0.27	1.37	0.49	1.08
	O2	0.44	1.25	1.81	1.82	1.10	1.33	1.39	1.02
	PO7	-0.74	1.43	0.53	1.77	-0.25	1.45	-0.01	1.15
	PO8	1.13	1.44	2.61	1.94	1.61	1.45	1.84	1.13
**N2pc latency**	O1/O2	314.09	5.85	324.71	7.11	340.82	8.42	342.77	7.06
	PO7/PO8	311.04	5.72	327.27	6.93	346.44	7.15	347.17	6.37

#### Accuracy

The overall accuracy for each participant was above 90%. A repeated measures analysis of variance (ANOVA) was conducted. Only a main effect of Orientation was observed, *F* (1, 15) = 8.37, *p* < .05, *η*_*p*_^*2*^ = .36, indicating that upright faces (*M* = 99.00%, *SE* = 0.66%) were detected more accurately than inverted faces (*M* = 98.41, *SE* = 0.70%). The main effect of Race and the interaction between Race and Orientation did not reach significance (*p*s >.17).

#### N170

**Fig 3:**
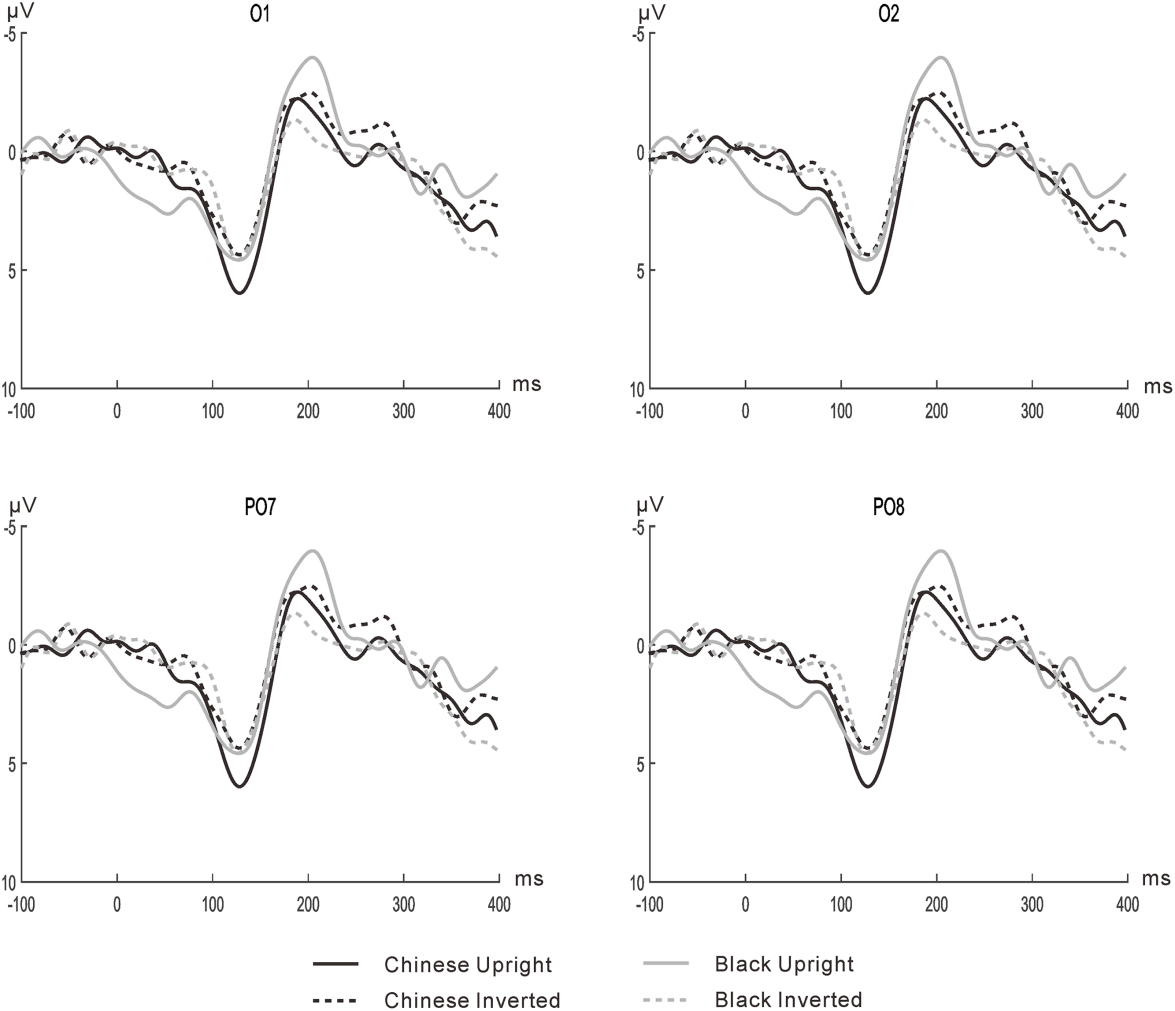
The waveforms for N170 and N250 at electrodes PO7/PO8 and O1/O2 in Experiment 2.

The mean amplitudes of N170 (see Fig 3) were entered into a four-way within-subjects ANOVA with the factors of Race (Black, Chinese), Orientation (upright, inverted), Hemisphere (left, right) and Electrode (O1/O2, PO7/PO8). Main effect of Hemisphere was significant, *F* (1, 15) = 6.45, *p* < .05, *η*_*p*_^*2*^ = .30, indicating that a reliable N170 effect was more negative for the left hemisphere than for the right hemisphere. The main effect of Electrode was significant, *F* (1, 15) = 8.30, *p* < .05, *η*_*p*_^*2*^ = .36, indicating a larger amplitude at electrodes PO7/PO8 than O1/O2. The main effect of Orientation reached marginal significance, *F* (1, 15) = 3.10, *p* = .099, *η*_*p*_^*2*^ = .17, with a stronger negative going for upright faces than inverted faces. Although the four-way interaction among Race, Orientation, Hemisphere and Electrode was significant, *F* (1, 15) = 6.07, *p* < .05, *η*_*p*_^*2*^ = .29, the analysis of separate Hemisphere or Electrode did not show any significant interaction of Race and Orientation (*ps* >.31). Other main effects and interactions did not reach significance (*p*s > .15).

#### N250

The four-way ANOVA for the mean amplitudes of N250 (see Fig 3) revealed a significant main effect of Hemisphere, *F* (1, 15) = 6.66, *p* < .05, *η*_*p*_^*2*^ = .31, indicating that a reliable N250 effect was more negative at left hemisphere than right hemisphere; and that there was a significant interaction of Hemisphere and Electrode, *F* (1, 15) = 7.56, *p* < .05, *η*_*p*_^*2*^ = .34. A simple effect analysis showed a significantly more negative effect for PO7 than PO8 (*p* = .01), but a marginally significant more negative effect for O1 than O2 (*p* = .06). Other main effects and interactions did not reach significance (*p*s > .14).

#### N2pc

**Fig 4:**
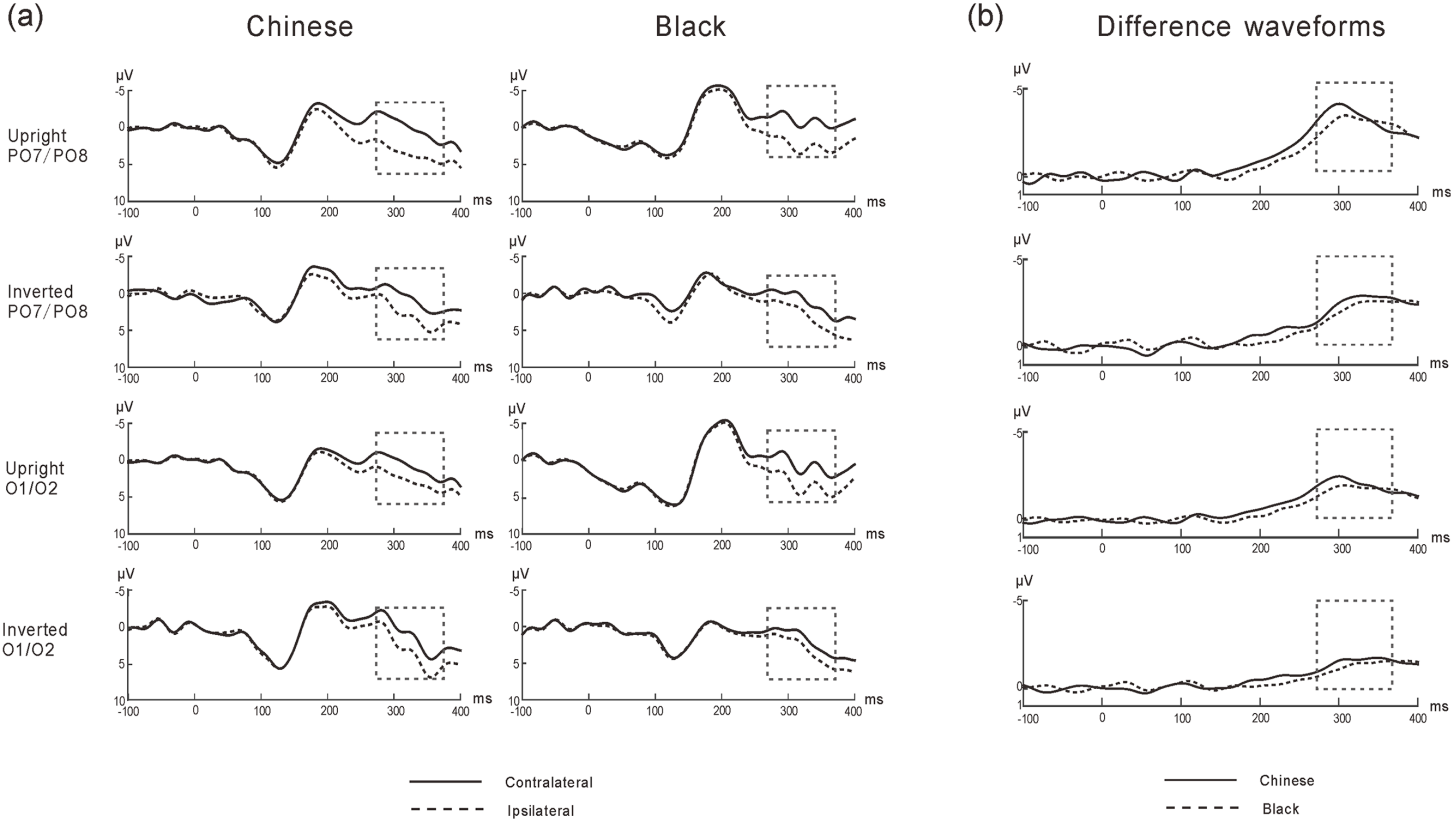
(a) Grand-averaged ERPs elicited by upright/inverted Chinese faces (left panel) and Black faces (right panel) at electrodes PO7/PO8, O1/O2 contralateral (solid lines) and ipsilateral (dashed lines) to the target in Experiment 2. (b) Difference waveforms of N2pc for upright/inverted Chinese faces (solid lines) and black (dashed lines) at electrodes PO7/PO8, O1/O2 in Experiment 2.

The N2pc wave was measured as the deflection recorded, contralateral to the visual field where the target appeared. Specifically, the N2pc amplitude is larger at electrode sites contralateral to the position of the target compared to ipsilateral sites. To quantify the peak latency of the N2pc wave, the averaged waveform in each condition recorded at the ipsilateral electrode sites was subtracted from the corresponding waveform recorded at the contralateral electrode sites at the occipital electrodes (i.e. when the target appeared in left visual field, PO8-PO7, O2-O1; when target appeared in right visual field, PO7-PO8, O1-O2). The N2pc wave can be seen as a more negative (i.e. less positive) voltage beginning at approximately 300 ms post-stimulus. The grand average ERP waveform is shown in Fig 4.

Within-subjects analysis of variance (ANOVA) with factors of Race (Black, Chinese), Orientation (upright, inverted) and Electrode (O1/O2, PO7/PO8) was employed for the N2pc latency. The ANOVA revealed a significant main effect of Race, *F* (1, 15) = 5.42, *p* < .05, *η*_*p*_^*2*^ = .27, indicating that the latency for Chinese faces (*Mean* = 328.09ms, *SE* = 5.84ms) was earlier than for Black faces (*Mean* = 335.48ms, *SE* = 5.22ms). The main effect of Orientation was significant, *F* (1, 15) = 18.84, *p* < .05, *η*_*p*_^*2*^ = .56, showing that upright faces evoked earlier N2pc waves compared with inverted faces. These results confirmed that own-race faces captured attention more quickly than other-race faces, and upright faces attracted attention more efficiently than inverted faces. The Race × Orientation interaction was not significant, *F* (1, 15) = 2.80, *p* = .12, *η*_*p*_^*2*^ = .16. These ERP results were consistent with the behavioral results (see Fig 4).

#### Correlation between RTs and N2pc latencies

**Fig 5:**
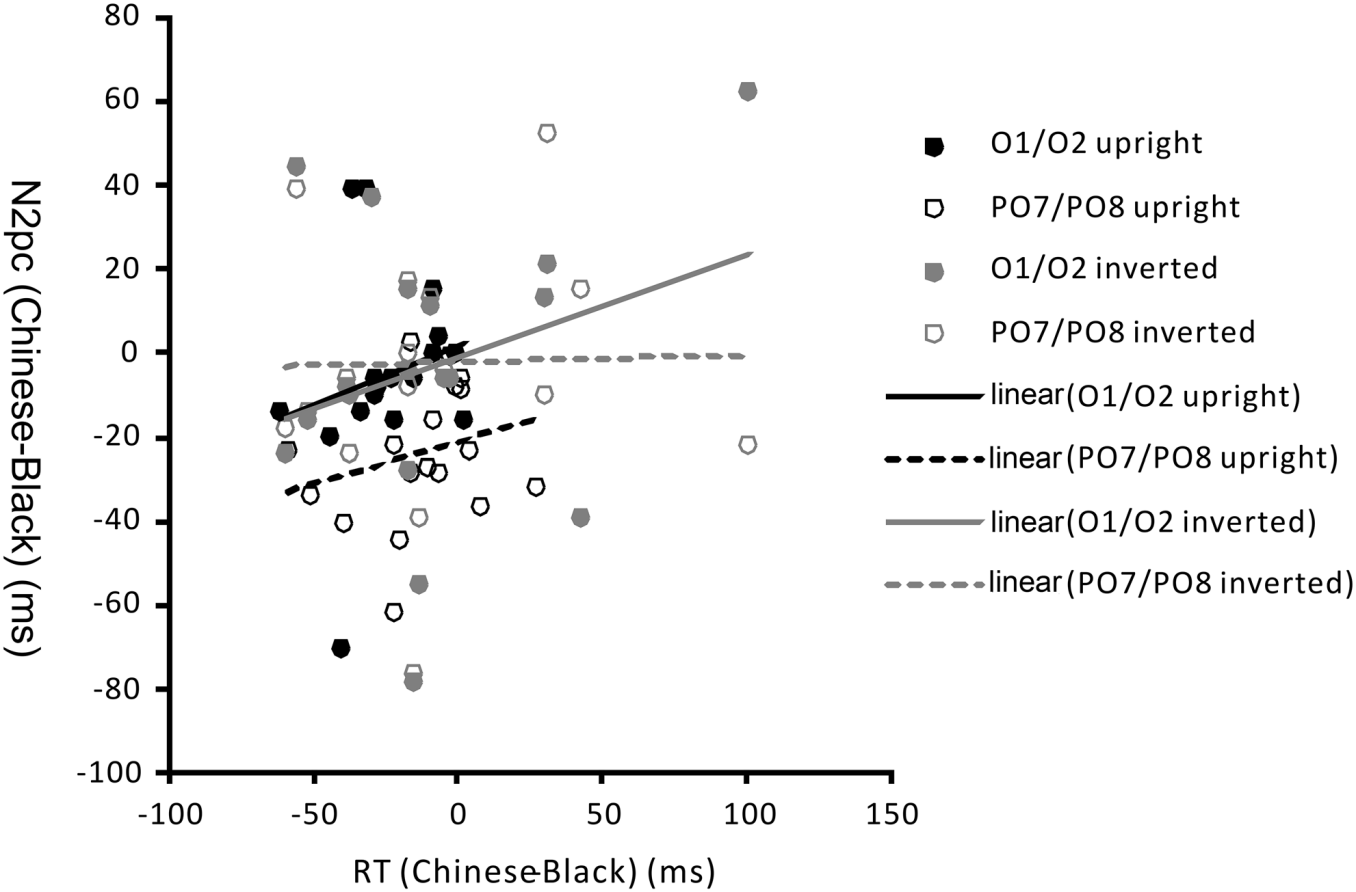
The correlation of RTs and N2pc latency in electrodes O1/O2, PO7/PO8 for upright faces and inverted faces in Experiment 2.

We computed Pearson correlations for the differences of Chinese and Black faces in response time (RT: Chinese-Black) with those in N2pc peak latency (N2pc latency: Chinese-Black) separately for the upright faces and the inverted faces. For upright faces, the difference in detection was marginally significantly correlated with the difference in the N2pc latency for electrode O1/O2, *r*(16) = .49, *p* = .06, and for PO7/PO8, *r*(16) = .45, *p* = .08. For inverted faces, their correlation was also marginally significant for electrode O1/O2, *r*(16) = .47, *p* = .07, but did not reach clear or marginal significance for PO7/PO8, *r*(16) = .13, *p* = .63. The tendency of this correlation implies that the larger the ORA in detection time, the earlier N2pc waves were evoked by own-race faces than other-race faces (see Fig 5). This result suggests that faster detection of own-race faces is related to faster attention being directed to them.

### Discussion

Experiment 2 replicated the ORA in RT. We attribute this ORA to attentional capture, which was confirmed by the earlier onset of N2pc waves for own-race faces than for other-race faces, and was further supported by the marginally significantly positive correlation between RTs and N2pc latencies of ORA size.

This Own-Race Advantage in behavior was not due to more configural processing for own-race faces than for other-race faces for two reasons. First, we did not observe a larger inversion effect for own-race faces than that for other-race faces in RT; N170 inversion effects were not observed for either own- or other-race faces. Second, own-race faces and other-race faces showed no difference in N170, which was regarded as an indicator of configural processing. This result was in line with previous research [32] [33] that used a race-irrelevant task, but inconsistent with other research [13] [31] that used a race categorization task; this suggests that the ORA in behavior is not related to race category processing. Moreover, this ORA in behavior is not due to more individual processing for own-race faces than for other-race faces, because no race difference was observed in N250.

We found an opposite (marginally significant) inversion effect for the N170, inconsistent with previous studies [27–29]. A possible reason is that human faces were presented intermixed with animal faces in the present study, since animal faces cannot elicit N170 amplitude inversion effects [41].

Although some previous studies have already found that own-race faces evoke a larger N2 wave, they used a categorization task [12–14] or an individual personality judgment task [13]. In addition, the posterior N2 is sensitive to different task-relevant visual features (a discrimination task), while the N2pc has been associated with the selection of the targets and the suppression of irrelevant distractors (a detection task) [42].

## General Discussion

In the present study, we found behavioral and ERP evidence that own-race and other-race faces are not processed equally even when no racial categorization or individuation is needed. The target human faces were detected faster when they were own-race faces than when they were other-race faces. More importantly, own-race faces elicited an earlier N2pc peak latency than other-race faces, which was correlated with faster detection. Those results indicate attentional superiority for own-race faces, consistent with previous results [5] [6], but inconsistent with the findings using a change blindness paradigm [8] [9] or a race-searching task [10] [11]. As we argued in the introduction, the discrepancy between these studies is due to the different processing levels required by the tasks. There is an other-race advantage in race category processing [10] [17] [18] and an own-race advantage in individuation processing [1] [18–20]. Therefore, the present study provides important evidence that there is an own-race advantage in the detection and differentiation of human/non-human faces.

The own-race advantage at the human/non-human level of face processing implies a preference for own-race faces in early perceptual processing. Specifically, an earlier N2pc in the present study was elicited in response to own-race faces than other-race faces among animal faces, demonstrating that the own-race faces can capture the attention more successful than other-race faces in a visual search array. This pattern of results could be explained from several aspects: Firstly, this advantage may be shaped by experience with own-race faces. That is, it may be due to the familiarity of stimuli. Previous studies show that target familiarity speeds up the allocation of visual-spatial attention [43] [44]. For example, Christie and Klein [43] used an attention allocation task to indicate whether the target position changed upward or downward, and their results showed better performance for word targets than non-word targets. Their findings demonstrate that familiar items may rapidly attract attention. Similarly, Chanon and Hopfinger [44] recorded participants’ eye movements when they viewed a scene with an old encoded target in it or the same scene with a new target in it. They found that old objects were fixated on significantly sooner than new objects, suggesting that attention could be exogenously drawn to the location of familiar items. Secondly, from an evolutionary perspective, because most interactions take place with in-group members, people are likely to be motivated to allocate attentional resources to own-race faces prior to maximize the expected utility (see [45] for a utilitarian theory account). Own-race faces can be regarded as high-valued stimuli. They have priority to be processed because valued stimuli can capture attention [46].

A limitation of the present study is that we did not explore whether the ORA in attention can explain the ORA in the traditional face memory task. However, recently researchers indeed found that participants spend more time on own-group faces than other-group faces during a self-paced face learning phase, and the individual differences in attention to own-group members during learning mediated superior memory for own-group members [47]. Divided attention during a face learning phase indeed impairs memory for own-race faces more than for other-race faces [48].

Another limitation of the present study is that we did not explore how attention interacts with some other processes in cross-race face processing, such as categorization or individualization. We suggest that attention varies with task demands: Categorization processes need more attention for other-race faces, and individual processing needs more attention for own-race faces. Further research is needed to resolve this issue.

In summary, the present study found an ORA in a human searching task with faster detection time, a shorter latency of N2pc for own-race faces than other-race faces. These results indicate that we have better performance for own-race faces than other-race faces in the early processing stage. Moreover, the present results provide electrophysiological evidence that own-race faces attract attention more efficiently than other-race faces.
